# Women’s Participation in Household Decision Making and Justification of Wife Beating: A Secondary Data Analysis from Pakistan’s Demographic and Health Survey

**DOI:** 10.3390/ijerph181910011

**Published:** 2021-09-23

**Authors:** Zohra S. Lassi, Anna Ali, Salima Meherali

**Affiliations:** 1 Robinson Research Institute, The University of Adelaide, Adelaide 5005, Australia; anna.ali@adelaide.edu.au; 2 Faculty of Nursing, University of Alberta, Edmonton, AB T6G 1C9, Canada; meherali@ualberta.ca

**Keywords:** women’s empowerment, decision making, wife beating, Pakistan, demographic and health survey

## Abstract

**Introduction:** Globally, women’s empowerment is one of the important factors impacting the development of the nation. However, several women in developing countries, including Pakistan, experience a high level of gender discrimination and inequity. In this study, data from the Demographic and Health Survey (DHS) were used to measure empowerment and its predictors among women in Pakistan. **Methods:** Pakistan’s 2017–2018 DHS dataset was used to measure women’s empowerment using two indicators, i.e., participation in decision making and views on wife beating among 4216 married women. The determinants of empowerment, such as age, place of residence, regions, wealth index, education, partner’s education, partner’s occupation, number of children, consanguinity, the age difference between husband and wife, house and land ownership, and house inheritance, are reported as prevalence ratios (PRs) with a 95% confidence intervals (CI). Multivariate regression models were used to produce covariate-adjusted PRs and 95% CIs. **Results:** More than half of all women were empowered (52.5%). Upon multivariate analysis, we identified that women from the province of Punjab (adjusted PR (aPR), 1.44; 95% CI, 1.20–1.73), Sindh (aPR, 1.62; 95% CI, 1.35–1.96), and KPK (aPR, 1.09; 95% CI, 0.91–1.31) compared to those living in Baluchistan; from the richest quantile (aPR, 1.65; 95% CI, 1.37–1.99), followed by the richer quantile (aPR, 1.54; 95% CI, 1.28–1.84), the middle quantile (aPR, 1.52; 95% CI, 1.28–1.81), and the poorer quantile (aPR, 1.24; 95% CI, 1.04–1.47) compared to women who were from the poorest quantile; who were highly educated (aPR, 1.45; 95% CI, 1.25–1.67), followed by those who had a secondary education (aPR, 1.32; 95% CI, 1.16–1.50) and a primary education (aPR, 1.17; 95% CI, 1.02–1.35) compared to women who were not educated; and had exposure to mass media (aPR, 1.20; 95% CI, 1.06–1.36) compared to those who had no exposure were more empowered. **Conclusion:** To conclude, women’s empowerment in Pakistan is affected by various socioeconomic factors, as well as exposure to mass media. Targeted strategies are needed to improve access to education, employment, and poverty alleviation among women, particularly those living in rural areas. Various mass media advertisements should be practiced, targeting community norms and supporting women’s empowerment.

## 1. Background

Empowerment in the context of gender has been defined as both a process and a result of a process that enables an individual to gain power, develop confidence, increase awareness, enhance mobility and choices, improve control over resources, and make decisions [1]. Empowerment takes shape from a context related to the social, cultural, economic, geographical, and political scenarios that a person experiences during their life course and their interaction with their gender roles in society. Empowerment helps in gaining opportunities and strength to voice opinions, accessing information, gaining exposure to new ideas and experiences, and taking responsibility to make decisions and risks. Women’s empowerment is essential to liberate women from the social, traditional, and cultural customs and norms and allows women to be aware of their rights, build self-confidence, have control over their and their significant others’ lives, and provide strength to bring change in society [2].

Gender inequality deeply prevails in developing countries, including Pakistan [3]. Of the total 149 countries in the world, Pakistan ranks the second lowest (148th) in gender equality, 146th in economic participation, and 97th in political empowerment [4]. In Pakistan, women’s empowerment has been neglected, and women have been deprived of their basic legal rights to have equal status and opportunity. Several factors are associated with women’s empowerment in Pakistan, including poverty and education [5]. According to the Demographic and Health Survey (DHS), women’s empowerment is indicated by participating in decision making, either alone or jointly with their husband, and disagreeing with all of the reasons that justify wife beating [6].

In Pakistani society, patriarchal setups, gender roles, and resulting violence have made women very vulnerable. The act of physical violence prevails due to deeply rooted sociocultural norms that have assigned gender roles for women. Wife beating/battering refers to violent acts that can be in the form of psychological, sexual, and/or physical assault by the husband against his wife with the intent to introduce fear and pain to control her [7]. In many parts of the world, this is a culturally and socially acceptable right that a husband has over his wife [8,9,10], and therefore, it significantly impacts her participation in decision making. Globally, approximately one in three women in their lifetime have been subjected to gender-based violence [11]. In Pakistan, most women are also secluded from public spaces because of their religious values, and therefore, their level of emancipation is assessed by participation in household decision making [12]. Men usually hold a dominant position in the family and are primarily responsible for supporting them financially. Women, on the other hand, are mostly homemakers, play a major role in childbearing, and provide nurturing and support in agricultural work, particularly in rural areas [13]. Moreover, in Pakistani society, gender has allocated roles, where men are generally the breadwinners of the family and women are caregivers; these gender allocation roles are more visible in rural areas compared to urban areas [14]. It is therefore important to assess the prevalence of women’s empowerment and its associated predictors in Pakistani society. A more recent study from Pakistan using the DHS dataset accounted for decision making and ownership for measuring empowerment reported that women who are in higher age brackets, educated, employed, residing in urban settings, heading the house, from wealthier quintiles, have children, and have access to information are more empowered [15]. Although the earlier study measured empowerment using ownership and decision-making indicators, this study considered decision making and wife beating as indicators to measure empowerment among women.

## 2. Methods

Pakistan’s Demographic and Health Survey (PDHS) has four datasets from 1990 to 2018. For the current study, we used the dataset of the year 2017–2018. Data from different regions of Pakistan were collected from ever-married women of reproductive age. In the current study, data on women’s empowerment were used. In the current dataset, information from 15,068 married women was collected, of which 4216 women responded to questions regarding women’s empowerment. Data regarding empowered women were obtained by secondary data analysis (SDA) of the recent DHS.

The outcome variable of this study was women’s empowerment, which was constructed by using the responses of four decision-making and five husband’s beating scenarios. The following questions were used to construct the outcome variables: Who decides (1) how to spend respondents earning?; (2) on the respondent’s healthcare purchases?; (3) on visits to family or relatives?; and (4) on spending husband’s earnings, as well as if beating justified if the respondent (1) goes out without telling her husband?; (2) neglects the children?; (3) argues with her husband?; (4) refuses to have sex with her husband?; and (5) burns the food? Empowered women were defined as “if the respondent or both the respondent and partner decided on the above-mentioned four decision-making questions, and if the respondent did not justify her husband’s beating related to the above five scenarios,” then these women were marked as empowered and the remaining were marked as unempowered women. The respondents’ age was merged into four categories, namely, <25, 25–34, 35–44, and 45 and above. The respondents’ and partners’ education were coded into four categories: No education, primary, secondary, and higher. The partners’ occupations were coded as not working, skilled, and unskilled. The respondents’ occupation was coded as working and not working. The place of residence was coded as urban and rural, while the region was coded into Punjab, Sindh, Khyber Pakhtunkhwa (KPK), and Baluchistan. Principal component analysis on assets ownership, including land and livestock, with a range of socioeconomic factors, including household construction, utilities, source of drinking water, and sanitation facilities, was used to construct a wealth index and categorized into five wealth quintiles: Poorest, poorer, middle, richer, and richest. Access to social media, consanguinity, land ownership, land inheritance, and had a say in choosing the husband were categorized as yes and no. If the respondent was exposed to the radio, newspapers, the Internet, and television, they were marked as access to media; otherwise, they were marked as no access to media. The number of children was categorized into none, less than 5, and more than or equal to 5. The age difference between husband and wife was categorized into the following categories: No difference, <5 years, 5–10 years, and more than 10 years.

Analysis was conducted in SPSS version 26 (IBM, Armonk, NY, USA). Categorical variables are reported as frequencies and percentages. Chi-square was used to report any differences between empowered and unempowered women. Bivariate associations between sociodemographics, media exposure, and land ownership were tested for statistical significance using Cox regression. All variables with borderline statistical significance (*p* < 0.25) were considered as potential confounding or interacting variables. The determinant of empowerment is reported as the prevalence ratio (PR) with a 95% confidence interval (CI). Multivariable regression models were used to produce covariate-adjusted PRs and 95% CIs. At this level, some categories were merged to avoid a small cell count problem. To select the final variables, we included all candidate variables (sociodemographic and media exposure) in the model and then applied purposeful backward elimination, until the model contained only variables significant at *p* < 0.05.

## 3. Results

**Figure 1:**
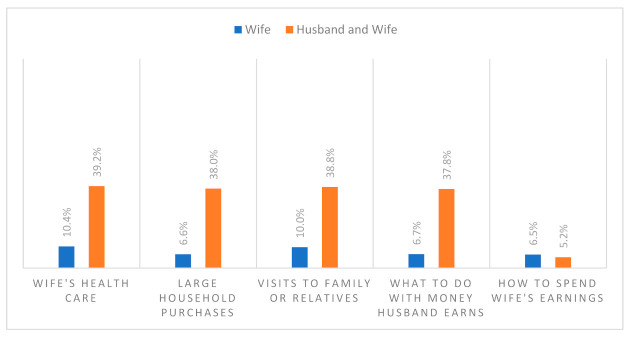
Women’s participation in decision making.

**Figure 2:**
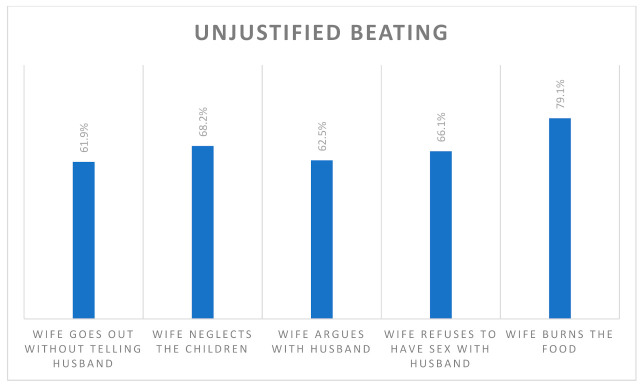
Wife beating was unjustified on different matters.

Of the total women included, data on women’s empowerment were available for 27.9% of the women, of which more than half were empowered (*n* = 2212, 52.5%). Figure 1 shows that 10% or fewer women participated in deciding on different matters on their own, while less than 40% participated in making a joint decision. Similarly, Figure 2 shows that more than 60% responded that wife beating is unjustified concerning different matters.

Of the total 4216 women, 39.8% were aged 25–35 years, while more than half were from rural areas (*n* = 2181, 51.7%), and the majority were from KPK (*n* = 1685, 40.0%), belonged to the poorest and poor wealth quantiles (*n* = 1785, 42.4%), were not educated (*n* = 2165, 51.4%), and were not working (*n* = 3542, 84.1%) (Table 1).

There was a significant difference between empowered and unempowered women with respect to age, place of residence, regions, wealth index, education, partner’s education, partner’s occupation, number of children, consanguinity, age difference between husband and wife, house and land ownership, and house inheritance. Empowered women were significantly younger (*p* < 0.001), were from urban areas (*n* = 1251, 56.6% vs. *n* = 784, 39.1%), were mostly from the regions of Sindh (*n* = 511, 23.1%) and Punjab (*n* = 829, 37.5%), and belonged to the richer (*n* = 491, 22.2%) and richest (*n* = 656, 29.7%) quantiles compared to unempowered women. Most of the empowered women and their partners were highly educated (women: *n* = 509, 23.0% vs. *n* = 125, 6.2%; husband: *n* = 675, 31.5% vs. *n* = 328, 16.9%) compared to unempowered women. Partners of empowered women were working professionally or engaging in skilled labor compared to unempowered women (*p* < 0.001). Empowered women had more prevalence of less than or equal to five children (*n* = 1625, 73.5%); however, unempowered women had more than five children (*n* = 529, 26.4%). Empowered women had a say in choosing a husband (*n* = 1813, 82.3% vs. *n* = 1529, 76.5%). The frequencies of land and house ownership and inheritance were very low; however, empowered women were significantly more likely to own and inherit houses and land compared to unempowered women (*p* = 0.004, 0.011, and <0.001, respectively) (Table 1).

Upon bivariate analysis, the PR for women’s empowerment was significantly higher among women aged 25–35 years (PR, 1.13; 95% CI, 1.01–1.28) and >45 years (PR, 1.22; 95% CI, 1.04–1.43). Women living in urban areas were more empowered compared to women of rural areas. Compared to Baluchistan, women from Punjab, Sindh, and KPK were significantly more empowered. Empowerment was significantly higher among women who were from the richest, richer, middle, and poor wealth quantiles compared to women who were from the poorest quantile. Empowerment was higher among women who were educated and also who had educated partners. Women with access to media were more empowered compared to those having no access to media. Likewise, women with consanguinity were significantly more empowered (PR, 1.17; 95% CI, 1.0–1.27). Women who had the right to choose their husband were more empowered compared to those who had not (PR, 1.19; 95% CI, 1.07–1.33). Lastly, women who owned or inherited a house or land were significantly more empowered compared to those who did not own or inherit a house or land (PR, 1.29; 95% CI, 1.09–1.53) (Table 2).

Upon multivariate analysis, regions, wealth index, respondent’s education, and media exposure were the significant predictors of women’s empowerment. The PR of women’s empowerment was significantly higher among women who were from the province of Punjab (PR, 1.44; 95% CI, 1.20–1.73), Sindh (PR, 1.62; 95% CI, 1.35–1.96), and KPK (PR, 1.09; 95% CI, 0.91–1.31) compared to those living in Baluchistan. Women’s empowerment was significantly higher among women who were from the richest quantile (aPR, 1.65; 95% CI, 1.37–1.99), followed by the richer quantile (aPR, 1.54; 95% CI, 1.28–1.84), the middle quantile (aPR, 1.52; 95% CI, 1.28–1.81), and the poorer quantile (aPR, 1.24; 95% CI, 1.04–1.47) compared to women who were from the poorest quantile. Women’s empowerment was higher for women who were highly educated (aPR, 1.45; 95% CI, 1.25–1.67), followed by those who had secondary education (aPR, 1.32; 95% CI, 1.16–1.50) and primary education (aPR, 1.17; 95% CI, 1.02–1.35) compared to women who were not educated. Lastly, women with media exposure were more empowered compared to those with no media exposure (aPR, 1.20; 95% CI, 1.06–1.36) (Table 2).

## 4. Discussion

This study presented findings on women’s empowerment in decision making and justification of wife beating using the 2017–18 PDHS dataset. The study findings revealed that almost half of women in Pakistan are not empowered and lack participation in household decision making. This study identified that women’s residing region, wealth index, education, and exposure to mass media are significantly related to their empowerment in Pakistan.

Women’s place of residence was significantly associated with their empowerment in Pakistan. The results highlighted that women living in Punjab, Sindh, and KPK were more empowered than women from Baluchistan. Baluchistan has mainly rural areas and its educational levels are lower than the rest of the country, and women there face a lack of economic opportunities that impact their decision making and empowerment. This study found a strong association between women’s education level and their empowerment. Women who were are highly educated were more empowered, as education enhances empowerment through increased skills, self-confidence, and knowledge [16,17]. Education serves as an enabler of empowerment and an avenue to autonomy [18], improves employment opportunities, makes decision making within the household more equal [18,19,20], and lessens the likelihood of endorsing gender-based violence [21,22,23].

The findings also highlighted that women who had a high household wealth index and women who owned and inherited a house and/or land were more empowered. Similar results have also been reported from Southeast Asian countries, showing that women in wealthier households are more likely to participate in decision making, jointly and on their own, than women of poorer households [15]. However, in Pakistan, women living in rural areas stand low on the wealth index [24], and also have inequitable access to family assets and inherited property that results in a lack of their influence and participation in decision making. Inequality and inability to own a house or land puts women in a precarious position, exacerbating poverty and unempowerment. In Pakistan, women’s right to inheritance is poorly realized, mainly due to patriarchal customs and sociocultural dynamics that give preference to men over women. In addition, women living in rural areas with a low level of education are not aware of their legal rights [15]. Hence, there is a dire need to introduce legal reforms and educate women about their legal rights in their parent’s or husband’s property. Such an affirmative action in policy and law could help to reduce gender inequality, enable women to own and inherit a house and land, and improve the socioeconomic status and health outcomes of women in Pakistan [25,26].

Lastly, our findings showed that women who were exposed to mass media were more empowered and had more equal gender role attitudes than those with no media exposure. Media exposure can help to empower women by equipping them with the information and means to function effectively, especially in the modern world [27]. Previous studies have revealed that media has a great potential for empowering women by increasing their participation and access to expression and decision making. Mass media is a powerful tool to promote mobilize women’s rights and challenge discrimination and other stereotypical behaviors against them [28,29]. Exposure to mass media also has the propensity to change norms, behaviors, and habits. For example, media can generate awareness about domestic violence and may be able to challenge gender stereotypes by portraying empowered and independent women, particularly among women with low education levels [30]. Studies from India and Bangladesh have found that television, radio, and/or print media influence gender norms related to violence against women [30,31,32].

This study had some limitations; first, the data were cross-sectional in nature and therefore could have affected the examination of temporality and any causal assumptions. A self-reported questionnaire was used to collect information on most of the variables, which therefore could have been subjected to recall bias. Despite these limitations, this study highlighted some of the significant determinants that impact women’s empowerment. The PDHS is the national representative survey; therefore, this study represents the prevalence and determinants of empowerment at the national level, meaning the findings are generalizable and could be used for devising policies for improving women’s empowerment at the national level.

## 5. Conclusions

The PDHS data analysis identified some important determinants that significantly impact women’s empowerment in Pakistan. Women’s empowerment is significantly influenced by women’s region of residence, wealth index, education, and media exposure. In light of these results, greater efforts are required to improve women’s access to educational and employment opportunities. Women’s education and employment are the areas identified as requiring gender-based equal opportunity initiatives through a policy to enhance the socioeconomic status of women and achieve development at the national scale. In addition, targeted actions are required to alleviate poverty among women living in rural areas, where women’s access to education, employment, and inheritance is mostly denied. Concerted efforts and collaborations are required from governments, human rights organizations, and civil societies to support and empower women living in rural and poor household wealth index areas. Moreover, legal frameworks are required to support women in owning or inheriting property. In addition, mass media should be used to communicate to women the relevance and ways of achieving women’s empowerment. Mass media should be used to educate both men and women about women’s rights and to change community norms and values that discriminate against women.

## Figures and Tables

**Table 1:** Sociodemographic characteristics of empowered women (*N* = 4216).

Sociodemographic Characteristics	Total Women *N* = 4216 (%)	Empowered *n* = 2212 (%)	Not Empowered *n* = 2004 (%)	*p*-Value
**Age**				
<25	719 (17.1)	339 (15.3)	380 (19.0)	0.003
25–35	1676 (39.8)	896 (40.5)	780 (38.9)	
35–45	1343 (31.9)	702 (31.7)	641 (32.0)	
>45	478 (11.3)	275 (12.4)	203 (10.1)	
**Place of residence**				
Urban	2035 (48.3)	1251 (56.6)	784 (39.1)	<0.001
Rural	2181 (51.7)	961 (43.4)	1220 (60.9)	
**Regions**				
Punjab	1279 (30.3)	829 (37.5)	450 (13.7)	<0.001
Sindh	786 (18.6)	511 (23.1)	275 (13.7)	
KPK	1685 (40.0)	712 (32.2)	973 (48.6)	
Baluchistan	466 (11.1)	160 (7.2)	306 (15.3)	
**Wealth Index**				
Poorest	813 (19.3)	244 (11.0)	569 (28.4)	<0.001
Poorer	972 (23.1)	377 (17.0)	595 (29.7)	
Middle	806 (19.1)	444 (20.1)	362 (18.1)	
Richer	772 (18.3)	491 (22.2)	281 (14.0)	
Richest	853 (20.2)	656 (29.7)	197 (9.8)	
**Education**				
No education	2165 (51.4)	817 (36.9)	1348 (67.3)	<0.001
Primary	578 (13.7)	322 (14.6)	256 (12.8)	
Secondary	839 (19.9)	564 (25.5)	275 (13.7)	
Higher	634 (15.0)	509 (23.0)	125 (6.2)	
**Partner’s education**				
No education	1145 (28.0)	431 (20.1)	714 (36.7)	<0.001
Primary	551 (13.5)	279 (13.0)	272 (14.0)	
Secondary	1390 (34.0)	758 (35.4)	632 (32.5)	
Higher	1003 (24.5)	675 (31.5)	328 (16.9)	
**Partner’s occupation**				
Not working	209 (5.1)	89 (4.2)	120 (6.2)	<0.001
Skilled	3007 (73.8)	1666 (78.1)	1341 (69.0)	
Unskilled	860 (21.1)	378 (17.7)	482 (24.8)	
**Occupation**				
Not working	3542 (84.1)	1858 (84.0)	1684 (84.1)	0.972
Working	672 (15.9)	353 (16.0)	319 (15.9)	
**Access to media**				
Yes	2775 (65.8)	1729 (78.2)	1046 (52.2)	0.307
No	1441 (34.2)	483 (21.8)	958 (47.8)	
**No. of children**				
0	456 (10.8)	240 (10.8)	216 (10.8)	<0.001
≤5	2884 (68.4)	1625 (73.5)	259 (62.8)	
>5	876 (20.8)	347 (15.7)	529 (26.4)	
**Consanguinity**				
Yes	2576 (61.1)	1266 (57.2)	1310 (65.4)	<0.001
No	1638 (38.9)	946 (42.8)	692 (34.6)	
**No. of co-wives**				
None	3964 (96.9)	2087 (97.4)	1877 (96.4)	0.057
>1	127 (3.1)	56 (2.6)	71 (3.6)	
**Age difference**				
None	264 (6.5)	158 (7.4)	106 (5.4)	0.048
<5 years	1486 (36.3)	752 (35.1)	734 (37.7)	
5–10 years	1500 (36.7)	791 (36.9)	709 (36.4)	
>10 years	840 (20.5)	443 (20.7)	397 (20.4)	
**Had a say in choosing the husband**				
Yes	3342 (79.6)	1813 (82.3)	1529 (76.5)	<0.001
No	859 (20.4)	389 (17.7)	470 (23.5)	
**Owns a house alone or jointly**				
Yes	128 (3.0)	83 (3.8)	45 (2.2)	0.004
No	4088 (97.0)	2129 (96.2)	1959 (97.8)	
**Owns land alone or jointly**				
Yes	105 (2.5)	68 (3.1)	37 (1.8)	0.011
No	4111 (97.5)	2144 (96.9)	1967 (98.2)	
**Inherit a house/land**				
Yes	111 (2.7)	88 (4.1)	23 (1.2)	<0.001
No	3979 (97.3)	2055 (95.9)	1924 (98.8)	

**Table 2:** Predictors of women’s empowerment.

Sociodemographic Characteristics	PR (95%CI)	aPR (95%CI)
Age *		
<25	Ref	
25–35	1.13 (1.01–1.28)	
35–45	1.09 (0.97–1.26)	
>45	1.221(1.04–1.043)	
Place of residence *		
Urban	1.39 (1.28–1.51)	
Rural	Ref	
Regions *		
Punjab	1.88 (1.59–2.23)	1.44 (1.20–1.73)
Sindh	1.89 (1.58–2.26)	1.62 (1.35–1.96)
KPK	1.23 (1.03–1.46)	1.09 (0.91–1.31)
Baluchistan	Ref	
Wealth Index *		
Poorest	Ref	
Poorer	1.29 (1.10–1.51)	1.24 (1.04–1.47)
Middle	1.83 (1.57–2.14)	1.52 (1.28–1.81)
Richer	2.11 (1.81–2.47)	1.54 (1.28–1.84)
Richest	2.56 (2.21–2.96)	1.65 (1.37–1.99)
Education *		
No education	Ref	
Primary	1.47 (1.29–1.67)	1.17 (1.02–1.35)
Secondary	1.78 (1.60–1.98)	1.32 (1.16–1.50)
Higher	2.12 (1.90–2.37)	1.45 (1.25–1.67)
Partner’s education *		
No education	Ref	
Primary	1.34 (1.15–1.56)	
Secondary	1.44 (1.28–1.63)	
Higher	1.78 (1.58–2.02)	
Partner’s occupation **		
Not working	Ref	
Skilled	1.03 (0.81–1.30)	
Unskilled	1.30 (1.05–1.61)	
**Occupation**		
Not working	Ref	
Working	1.00 (0.89–1.12)	
Access to media *		
Yes	1.85 (1.68–2.05)	1.20 (1.06–1.36)
No	Ref	Ref
No. of children *		
0	Ref	
≤5	1.07 (0.93–1.22)	
>5	0.75 (0.63–0.88)	
Consanguinity *		
Yes	1.17 (1.08–1.27)	
No	Ref	
No. of co-wives **		
None	1.19 (0.91–1.55)	
>1	Ref	
**Age difference**		
None	1.13 (0.94–1.36)	
<5 years	0.96 (0.85–1.07)	
5–10 years	1.00 (0.89–1.12)	
>10 years	Ref	
Had a say in choosing the husband *		
Yes	1.19 (1.07–1.33)	
No	Ref	
Own or inherited land/house *		
Yes	1.29 (1.09–1.53)	
No	Ref	

* *p* < 0.001 and ** *p* < 0.25 upon univariate analysis.

## Data Availability

Data availability: The data used in this study were from the individual recode data file of the Pakistan 2019 Demographic and Health Survey, available from the Demographic and Health Survey (DHS) website. Consent was obtained from the participants included in the survey, and the data provided were de-identified. Access to the dataset requires registration and is granted only for legitimate research purposes. A guide for how to apply for dataset access is available at: https://dhsprogram.com/data/Access-Instructions.cfm (accessed on 10 June 2021).

## References

[1] Raj S., Ravi R.V., Latha H.M.H. (2010). Women, and empowerment: A perspective. In Women in Development: Challenges and Achievements.

[2] Sohail M. (2014). Women empowerment and economic development-an exploratory study in Pakistan. J. Bus. Stud. Q..

[3] Raza A., Murad H.S. (2010). Gender gap in Pakistan: A socio-demographic analysis. Int. J. Soc. Econ..

[4] Masitoh D., Pramesti F.A. (2020). Gender Inequality in Pakistan Caused by Domestic Factors and Conflict Resolving Based on CEDAW. Nation State: J. Int. Stud..

[5] Bushra A., Wajiha N. (2015). Assessing the socio-economic determinants of women empowerment in Pakistan. Procedia-Soc. Behav. Sci..

[6] Kishor S., Subaiya L. (2008). Understanding Women’s Empowerment: A Comparative Analysis of Demographic and Health Surveys (DHS) Data.

[7] Herbert C.P. (1983). Wife battering. Can. Fam. Physician.

[8] Jejeebhoy S.J. (1998). Wife-beating in rural India: A husband’s right? Evidence from survey data. Econ. Political Wkly..

[9] Uthman O.A., Lawoko S., Moradi T. (2009). Factors associated with attitudes towards intimate partner violence against women: A comparative analysis of 17 sub-Saharan countries. BMC Int. Health Hum. Rights.

[10] Seidu A.-A., Dzantor S., Sambah F., Ahinkorah B.O., Ameyaw E.K. (2021). Participation in household decision making and justification of wife beating: Evidence from the 2018 Mali Demographic and Health Survey. Int. Health.

[11] WHO (2021). Devastatingly Pervasive: 1 in 3 Women Globally Experience Violence.

[12] Mujahid-Mukhtar E., Mukhtar H., Abbink G.A. (1991). Female Participation in Household Decision-making: An Analysis of Consumer Durables’ Acquisition in Pakistan [with Comments]. Pak. Dev. Rev..

[13] Mahmood N. (2002). Women’s role in domestic decision-making in Pakistan: Implications for reproductive behaviour. Pak. Dev. Rev..

[14] Sarwar A., Imran M.K. (2019). Exploring Women’s multi-level career prospects in Pakistan: Barriers, interventions, and outcomes. Front. Psychol..

[15] Abbas S., Isaac N., Zia M., Zakar R., Fischer F. (2021). Determinants of women’s empowerment in Pakistan: Evidence from Demographic and Health Surveys, 2012–2013 and 2017–18. BMC Public Health.

[16] Cornwall A. (2016). Women’s empowerment: What works?. J. Int. Dev..

[17] Klugman J., Hanmer L., Twigg S., Hasan T., McCleary-Sills J., Santamaria J. (2014). Voice and Agency: Empowering Women and Girls for Shared Prosperity; World Bank Publications. https://openknowledge.worldbank.org/handle/10986/19036License:CCBY3.0IGO.

[18] Albert C., Escardíbul J.O. (2017). Education and the empowerment of women in household decision-making in S pain. Int. J. Consum. Stud..

[19] Head S.K., Yount K.M., Hennink M.M., Sterk C.E. (2015). Customary and contemporary resources for women’s empowerment in Bangladesh. Dev. Pract..

[20] Salem R., Cheong Y.F., Yount K.M. (2018). Is women’s work a pathway to their agency in rural Minya, Egypt?. Soc. Indic. Res..

[21] Doku D.T., Asante K.O. (2015). Women’s approval of domestic physical violence against wives: Analysis of the Ghana demographic and health survey. BMC Women’s Health.

[22] Conroy A.A. (2014). Gender, power, and intimate partner violence: A study on couples from rural Malawi. J. Interpers. Violence.

[23] Rashid M., Kader M., Perera N.K., Sharma A. (2014). Wife beating: A population-based study in Bangladesh. Violence Gend..

[24] Phan L. (2016). Measuring women’s empowerment at household level using DHS data of four Southeast Asian countries. Soc. Indic. Res..

[25] Ekhator E.O. (2015). Women and the law in Nigeria: A reappraisal. J. Int. Women’s Stud..

[26] Lukito R. (2006). The Enigma of National Law in Indonesia: The Supreme Court’s Decisions on Gender-Neutral Inheritance. J. Legal Pluralism Unofficial Law.

[27] Seidu A.-A., Ahinkorah B.O., Hagan J.E., Ameyaw E.K., Abodey E., Odoi A., Agbaglo E., Sambah F., Tackie V., Schack T. (2020). Mass media exposure and women’s household decision-making capacity in 30 sub-Saharan African countries: Analysis of demographic and health surveys. Front. Psychol..

[28] Narayana A., Ahamad T. (2016). Role of media in accelerating women empowerment. Int. J. Adv. Educ. Res..

[29] Loiseau E., Nowacka K. (2015). Can social media effectively include women’s voices in decision-making processes. Paris: Organ. Econ. Coop. Dev..

[30] Jesmin S.S., Amin I. (2017). Impact of the mass media in changing attitudes towards violence against women in Bangladesh: Findings from a national survey. J. Fam. Violence.

[31] Bhattacharya H. (2016). Mass media exposure and attitude towards spousal violence in India. Soc. Sci. J..

[32] Bhushan K., Singh P. (2014). The effect of media on domestic violence norms: Evidence from India. Econ. Peace Secur. J..

